# Comparative Approach to De-Noising TEMPEST Video Frames

**DOI:** 10.3390/s24196292

**Published:** 2024-09-28

**Authors:** Alexandru Mădălin Vizitiu, Marius Alexandru Sandu, Lidia Dobrescu, Adrian Focșa, Cristian Constantin Molder

**Affiliations:** 1 Faculty of Electronics, Telecommunications and Information Technology, National University of Sciences and Technologies Politehnica Bucharest, 060042 Bucharest, Romania; lidia.dobrescu@upb.ro; 2 The Special Telecommunications Service, 060044 Bucharest, Romania; marius.sandu@stsnet.ro; 3 Center of Excellence in Robotics and Autonomous Systems—CERAS, Military Technical Academy “Ferdinand I”, 050141 Bucharest, Romania; adrian.focsa@mta.ro (A.F.); cristian.molder@mta.ro (C.C.M.)

**Keywords:** TEMPEST, security, de-noising, adaptive Wiener filter, U-Net, CNN

## Abstract

Analysis of unintended compromising emissions from Video Display Units (VDUs) is an important topic in research communities. This paper examines the feasibility of recovering the information displayed on the monitor from reconstructed video frames. The study holds particular significance for our understanding of security vulnerabilities associated with the electromagnetic radiation of digital displays. Considering the amount of noise that reconstructed TEMPEST video frames have, the work in this paper focuses on two different approaches to de-noising images for efficient optical character recognition. First, an Adaptive Wiener Filter (AWF) with adaptive window size implemented in the spatial domain was tested, and then a Convolutional Neural Network (CNN) with an encoder–decoder structure that follows both classical auto-encoder model architecture and U-Net architecture (auto-encoder with skip connections). These two techniques resulted in an improvement of more than two times on the Structural Similarity Index Metric (SSIM) for AWF and up to four times for the SSIM for the Deep Learning (DL) approach. In addition, to validate the results, the possibility of text recovery from processed noisy frames was studied using a state-of-the-art Tesseract Optical Character Recognition (OCR) engine. The present work aims to bring to attention the security importance of this topic and the non-negligible character of VDU information leakages.

## 1. Introduction

The threat posed by unintended emanations in the video domain has drawn attention from the research community since W. Van Eck’s publication on compromising emanations from Cathode Ray Tube (CRT) screens in 1985 [1]. The author showed that not only is this type of attack possible but that it can be accomplished using cost-efficient equipment. The following investigations [2,3] have contributed to a better understanding of the phenomenon. The author details in [2] the threat posed by electromagnetic radiation from RS-232 cables, and Markus G. Kuhn extended the existent work from only studying CRT’s emanations to documenting eavesdropping experiments on flat panel displays [3] in 2003. Others [4,5,6] continued the research in this particular field and have solidified the concept that video display units in general but especially video data signaling, like HDMI, Video Graphics Array (VGA), Display Visual Interface (DVI), and Low-Voltage Differential Signaling (LVDS), suffer from compromising electromagnetic emanations. An important change in this research field has happened since the development of Software-Defined Radios (SDRs). This radio equipment has features that ease the TEMPEST signals’ recovery process. Together with the open-source available software TempestSDR v1 [7], exploiting compromising emanations of VDUs has become more accessible. Although this type of attack still may resemble an intelligence method from the Cold War era, data theft through eavesdropping can be a serious security challenge because of its undetectable nature. In [8], the authors explore the possibility of reconstructing video frames from a distance of 80 m under realistic circumstances. As experimental results show in [8], the reconstructed video frames have a considerable amount of noise. Additionally, their quality could be decreased by any other possible interference in the specific environment. To quantitatively analyze the security issue created by VDU leakages, it is necessary to assess whether the reconstructed frames can be integrated into an automated information extraction system, represented by optical character recognition engines, which would automatically process the frames and extract the information displayed on the target monitor as a text file. OCR tools, implemented with machine learning algorithms, are very popular and aim to extract machine-coded text from digital images. For the present work, the Tesseract OCR [9] engine was used, with no preliminary results on text extraction for noisy reconstructed frames. 

This paper aims to de-noise the video frames to improve image quality and to make possible optical character recognition for the reconstructed digital images. To achieve this goal, two methods were implemented and tested. The first technique, the adaptive Wiener filter, is implemented in the spatial domain and utilizes a sliding window to modify the image. This type of filter adjusts its performance based on local statistical parameters along the image, assuming that areas with high local variance represent important details and edges that need to be preserved. It exhibits linear behavior, acting as a mean filter for the areas with a small local variance while keeping the original values unchanged for the areas with edges and details. A detailed comparative analysis regarding both the filter versions in the spatial domain and the frequency domain may be found in [10], where the advantages of using adaptive image processing techniques in protecting sensitive areas have also been highlighted.

The second approach involves modern deep learning algorithms, specifically, Convolutional Neural Networks (CNNs) [11,12,13], to implement a network with an encoder–decoder structure. The objective is to learn a latent representation of the noisy video frame and reconstruct its corresponding clean version. The model architecture follows a U-Net structure, featuring two symmetric CNNs with skip connections between them. DL algorithms have been proven to be very useful in the context of TEMPEST images, and several successful experiments have already been published [14,15]. The authors detail in [14] the training process of a deep convolutional encoder–decoder network to remove white Gaussian noise, yielding promising results. Also, closer to our subject, ref. [15] explores various artificial intelligence approaches for its goal of de-noising intercepted frames, including U-Net, auto-encoder, and Mask R-CNN. 

Other state-of-the art de-noising techniques rely on transformer-based networks, and J. Liang et al. [16] performed successful experiments in video restoration by incorporating recurrent design into transformer-based models.

The authors of [17] propose a different approach in this field, as their method consists of first processing the image with a small set of highly specialized de-noisers, each trained with a different noise distribution and then passing their results to a kernel prediction network that estimates per-pixel de-noising kernels. The method is well suited for real-world applications where the noise distribution is unknown. The main difference between the other works presented and our approach lies in the information security analysis on the text in the de-noised video frames.

The proposed methods in this paper were assessed through the SSIM. The SSIM was first introduced in [18], and it relies on extracting three key features from an image: luminance, contrast, and structure. A comparison between two images is made considering these three features. Also, for evaluating the performance, OCR precision on de-noised frames was analyzed and discussed.

## 2. Materials and Methods

### 2.1. Data Acquisition Setup

Considering that the phenomena of TEMPEST compromised signal emanations is well known in the communication security field, the work in this paper is focused on laboratory measurements since the main purpose of the experiment is to employ new methods for improving video raster. Distances or environmental conditions could affect the data acquisition process unless an adequate preamplifier is used in the receiver chain, and thus they must be carefully considered for real-scenario experiments. 

**Figure 1:**
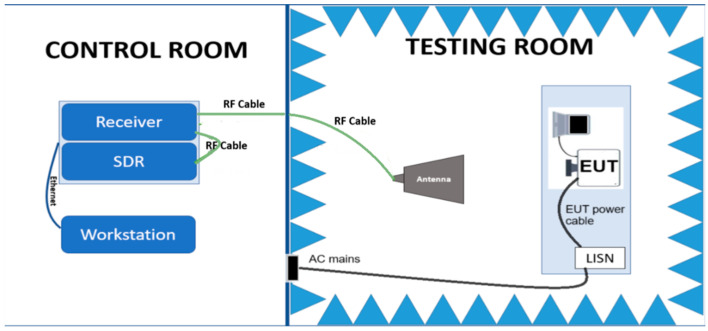
The setup used to capture EUT’s emanations.

Unintended emanations were captured in a laboratory setup, as shown in Figure 1, using an anechoic chamber, which ensures a clean electromagnetic environment and a control room to process the leaked video signal. The equipment used was a 1980 × 1080 resolution Dell S2421HN LED monitor with a 60 Hz refresh rate and a pixel clock value equal to 148,500 MHz. It was placed on the test bench and connected to the graphics card of a TEMPEST pre-tested laptop to prevent unwanted interferences. Equipment Under Test (EUT) is connected to the power supply through a Line Impedance Stabilization Network (LISN), which filters any radio frequency signal that may appear as a conducted emission from the AC main and could affect the experiment’s quality and creates a known impedance between the EUT and power line providing the conducted emission testing.

For the reception of compromising radiation emanations corresponding to the video signal generated by the studied equipment, appropriate electromagnetic translators suitable for the spectrum of interest were used. Thus, in the frequency range of 30 MHz–200 MHz, the R&S HE526 active antenna was used, while in the frequency range of 200 MHz–1 GHz, the R&S HE527 active antenna was used. The signal received with the help of a translator was then transferred to the control room where the reception equipment was located through an RF connector panel. The reception equipment consisted of a radio frequency receiver, an SDR, and a workstation for controlling the equipment and processing the received signals. The employed receiver was a wideband receiver suitable for TEMPEST testing and evaluation, R&S FSWT26.

**Figure 2:**
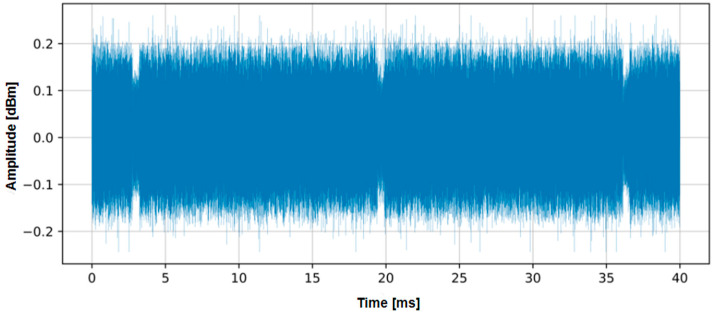
Captured video signal visualized in the time domain.

The acquisition of the video signal was undertaken using an SDR SignalHound BB60D, and Figure 2 displays two complete video frames with a total duration of 33.4 ms.

Despite the availability of a spectrum receiver equipped with an image reconstruction (raster) module, a more accessible and user-friendly application was developed to meet the specific requirements of the task. The principle of image reconstruction from unintended electromagnetic radiation is well known and has been extensively studied since the late 20th century. Currently, the literature does not describe a method for obtaining colored images from the analyzed device’s emissions. Instead, colored images are obtained using various gradient techniques to represent pixel values.

Regarding video frame composition and its parameters, the process of image reconstruction from video display units involves scaling the captured samples within a range of 0–255, resulting in grayscale image reconstruction. Considering the video frame refresh rate and the display’s resolution, the 1D array was reshaped into a 2D array. Applying a median filter to the resulting matrix facilitated the reconstruction of the video frame corresponding to the image displayed by the analyzed device.

**Figure 3:**
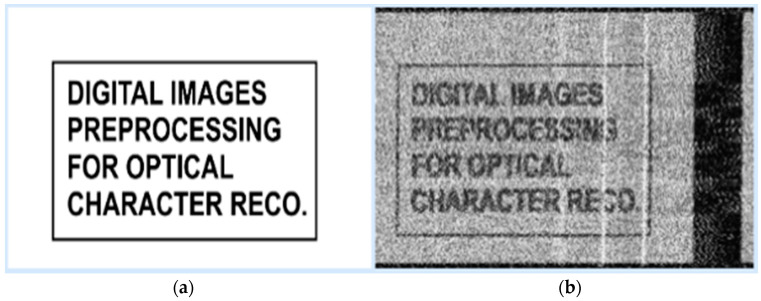
(**a**) Image to be displayed on the test monitor; (**b**) reconstructed image after processing the video signal.

Due to the video signal’s bandwidth and the unintentional generation of harmonics by the studied device, the image reconstruction process initially produced a noisy frame. To improve the clarity of the obtained image, a median filter was applied. Those frames served as input data for this experiment. It is worth noticing that determining the video line duration precisely is very important for a successful reconstruction process. In Figure 3, one test image is presented in both its clean version and its reconstructed state. 

### 2.2. Adaptive Wiener Filter

Images studied in the present paper are very specific, featuring a white background with black characters. The objective of a conventional filtering method is to preserve the characters while effectively smoothing as much as possible the background, enhancing the signal-to-noise ratio (SNR) value. For this task, the adaptive Wiener filter implemented in the spatial domain was considered the most appropriate. Its main theoretical purpose is to estimate the image without noise, minimizing mean square error between noisy data and ground truth by using local statistics. To implement the filter using the pixel-by-pixel method, a sliding window is used, within which, based on local statistical parameters, the corrected value of the central pixel is determined according to the linear combination between the local mean value and the unmodified pixel value. Applying this logic, the weight given by the average of neighboring pixels and that given by the noisy image itself were determined by the ratio between the local variance in the window and the maximum local variance considering windows with the same size, as described in Equation (1):

(1) $$r\left(i,j\right)=\alpha \cdot µ(i,j)+\beta \cdot s(i,j),$$

where

(2) $$\alpha =\frac{\sigma_{max}-\sigma_{L}(i,j)}{\sigma_{max}}$$

and

(3) $$\beta =\frac{\sigma_{L}(i,j)}{\sigma_{max}}$$

with $\sigma_{L}(i,j)$ being the local variance in that particular window, $\sigma_{max}$ the maximum local variance along the image (taking into consideration windows with the same size only), r(i,j) the modified pixel in the filtered image, and s(i,j) the original pixel. This strategy assures a simple linear model that processes the input noisy frames accordingly for each area in the image. For the purpose of further explanations, the ratio between $\sigma_{L}(i,j)$ and $\sigma_{max}$ will be called the relative local variance in this paper.

**Figure 4:**
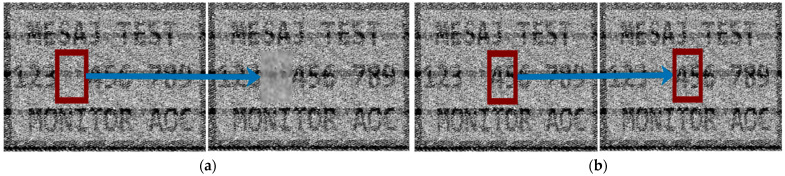
(**a**) Filter effectively applies a mean filter in the area selected; (**b**) the area selected has high local variance and r(i,j)’s value is mostly influenced by s(i,j).

The immediate question that needs to be addressed is how large should the window be. First, if considering windows with small sizes (3 × 3, 5 × 5, 7 × 7), the changes will be visible but not quite satisfactory. Nevertheless, this could be useful in many cases. Secondly, if larger windows are used, an effective visible blur will be caused in the background, but closer to the characters, a large local variance value will be computed within the window, and no modifications will be made to the pixels in characters’ proximity. The latter is presented in Figure 4. The unfiltered noise around the characters could pose a problem for the Tesseract OCR engine, which uses a binarization algorithm as a preprocessing step before character prediction. 

**Figure 5:**
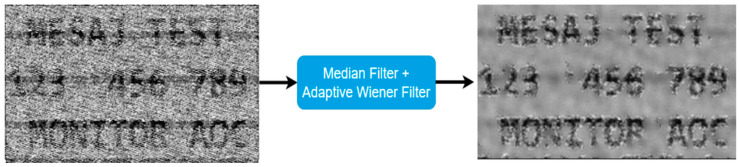
Final visual result for adaptive Wiener filter approach; it should be noted that a median filter was used before the proposed method.

Adaptive window size was implemented in order to fix the issue presented. Specifically, by default, a large window will be set (e.g., 13 × 13, 15 × 15, 17 × 17). However, whenever the relative local variance exceeds a threshold value within that large window that has been set by default, the window size will automatically be adjusted to 3 × 3 as it is assumed that a sensitive area is being handled at that moment, and all the operations that define this filter will be performed using 3 × 3 windows. Also, whenever the relative local variance value decreases under the threshold, filtering process will be handled using the large window. By incorporating this feature into the adaptive Wiener filter, it is possible to benefit both from efficient background blur produced caused by large windows and from the heightened attention paid by small windows in areas closely adjacent to the characters. The result of applying this method is presented in Figure 5.

For the present work, the filter was applied to images 512 × 256 in size, the large window size was 13 × 13, and the threshold value was set to 0.85. For reproducibility, it is recommended to maintain the same proportions between the image size and the default window size. 

### 2.3. Deep Learning

The recent results of artificial intelligence in various domains recommend these methods for widespread use and may represent a solution for the abstract nature of the noise in TEMPEST reconstructed images.

**Figure 6:**
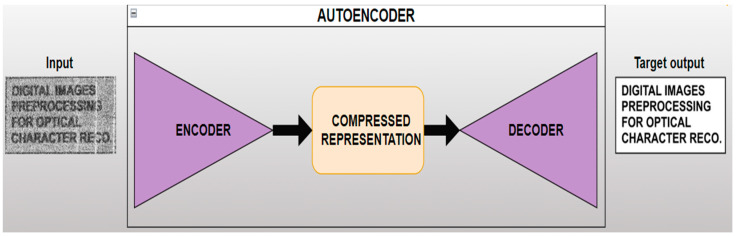
Visual summary of the proposed deep learning method.

The second proposed approach involved utilizing the auto-encoder-based DL method for this de-noising task. An auto-encoder is built from two CNNs with symmetric architecture and a central latent representation called a bottleneck. The first CNN, known as the encoder, performs basic operations specific to feature extraction. The output of the encoder is a compressed representation of data capturing important features. The decoder then attempts to up-sample the latter in order to reconstruct input data. In this paper, the implementation of the auto-encoder aimed to use the noisy video frames as input and force the model to reconstruct them into clean versions, as shown in Figure 6. 

#### 2.3.1. Creating the Dataset

**Figure 7:**
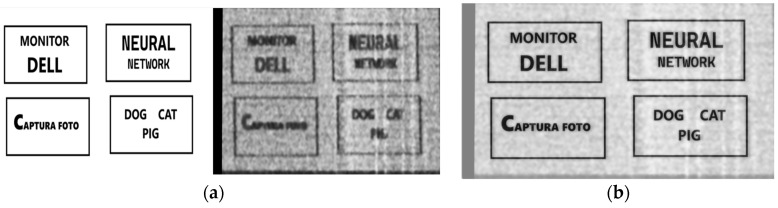
(**a**) Image alignment stacked; (**b**) image alignment overlay.

One of the main challenges is creating the dataset because for each noisy video frame, its corresponding clean version is needed in order to be used as a target for the model prediction in the training phase. Thus, the dataset will consist of several reconstructed–original image pairs. As presented in Figure 3, the intercepted frame is skewed, noisy, and misaligned. Additionally, synchronization errors could be observed, causing image displacement. Knowing that the loss between model prediction and the original image has to be computed, an exact alignment has to exist. To create the dataset and to overcome the challenges presented, on the test monitor four patches of text were displayed, and the reconstructed frame was aligned with its corresponding clean version using the Oriented FAST and Rotated BRIEF (ORB) algorithm. Its performance was evaluated and compared to other state-of-the-art algorithms, like SIFT and SURF in [19]. The result of applying ORB algorithm to align noisy video frames can be seen in Figure 7.

The alignment was not always possible unless supplementary filtering was applied to the TEMPEST video frames. These filters include the median filter and the adaptive Wiener filter detailed above in this paper. The alignment was verified through overlaying the image pairs, and in the cases where the ORB still could not produce satisfactory results after applying the filters, the text patches were not included in the dataset in order to avoid compromising the experiment. Nevertheless, biases or errors that could result after applying ORB might be compensated by integrating a loss function that is invariant to misalignment errors, such as Normalized Compressed Distance (NCD) [20] or the SSIM. 

**Figure 8:**
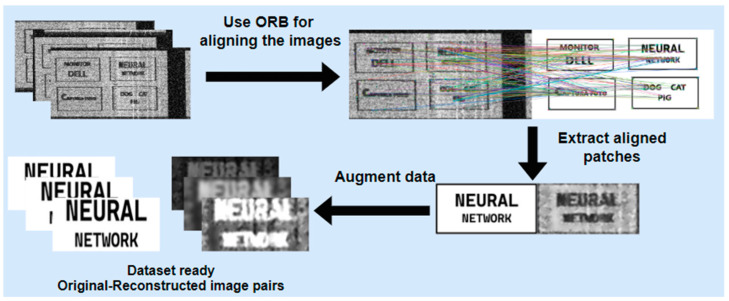
The approach for creating the dataset.

Once the reconstructed frame is aligned with the clean one, each patch of text is extracted in pairs, and several augmentation operations are applied to increase the variability of the dataset. Specifically, random Gaussian noise, random Gaussian blur, random contrast adjustments, random brightness adjustments, random cropping, and histogram equalization are applied. The entire processing flow is schematically depicted in Figure 8. 

The proposed approach generated 4000 image pairs which were split into training data and validation data at a ratio of 4 to 1. The augmentation ensured diversity throughout the dataset by applying various transformations in order to reduce the risk of overfitting and to allow the model to generalize better to new data. Table 1 notes the percentage of the dataset to which a type of augmentation has been applied.

#### 2.3.2. Training and Models

The training process was conducted using a GTX 1080 graphics card with 12 GB of memory. The input images’ dimension was 512 × 256 in grayscale format. The feature extraction block (encoder) consisted of convolutional layers with an increasing number of filters and a 3 × 3 kernel size. The compressed data representation had a dimension of 32 × 16 × 128 and served as input for the decoder block which utilizes transpose convolutional layers to reconstruct the image. For the whole network, regularization techniques, such as dropout and batch normalization, were used to prevent overfitting during training. Also, as activation function for the training Rectified Linear Unit (ReLu) was employed, while at the last convolutional layer, sigmoid was used. The deep learning model was trained for 50 epochs, with a batch size of 32 and a learning rate 0.05, utilizing Mean Squared Error (MSE), described in Equation (4) as the loss function:

(4) $$MSE=\frac{\sum_{k=1}^{N}{\left\|Y_{k}-\hat{Y_{k}}\right\|}^{2}}{N}$$

where $Y_{k}$ is the predicted value for the k-th sample, $\hat{Y_{k}}$ is the target value for the k-th sample, and N is the number of samples in the dataset. In order to update the weights’ values, an Adam optimizer was utilized.

**Figure 9:**
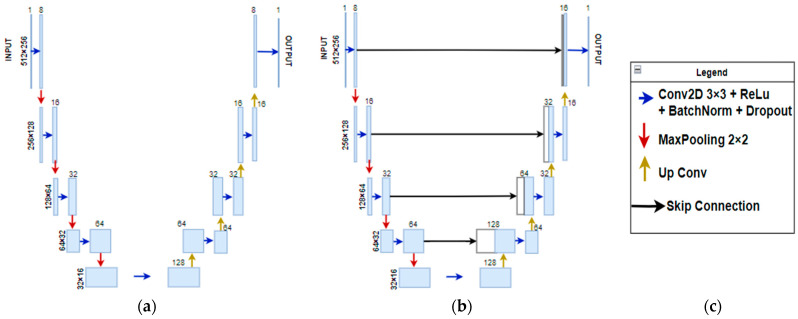
(**a**) Basic auto-encoder architecture; (**b**) auto-encoder with skip connections (U-Net); (**c**) legend.

In order to achieve optimal results, both the classical auto-encoder architecture discussed earlier and the U-Net architecture, which is a specific implementation of auto-encoder that involves adding skip connections between the symmetrically disposed layers, were tested. U-NET is very well known for image segmentation [21], but can also be used for de-noising tasks. The two model architectures are schematically described in Figure 9, together with data dimensions and the number of filters at every step of the network.

Initial experiments showed that the number of filters was an important hyperparameter for this application because having too many neurons not only increased training time but also generated overfitting issues. In the final network configuration, the number of filters ranged from 1 to 128 as the network became deeper. Several tests also focused on the behavior of the model when ranging the learning rate from 0.005 to 0.5, and the conclusion was that the model’s capability to convergence was not affected but only the time it took to converge.

The addition of skip connections to the encoder–decoder structure was inspired by deep Residual Network (ResNet) models [22], and the benefit, in the case of this experiment, addresses two possible drawbacks of the classical auto-encoder. During the feature extraction process, important image details can be lost, making efficient reconstruction much more challenging. When skip connections are part of the architecture, the feature maps passed to deeper layers carry important image details, which can be helpful for deconvolution layers to reconstruct a much cleaner image. Additionally, a problem frequently addressed in the literature is that deeper networks could suffer from gradients vanishing, making it difficult for the networks to learn efficiently. Skip connections tackle this issue by creating additional paths for the gradient flow, allowing for better backpropagation through the network.

**Figure 10:**
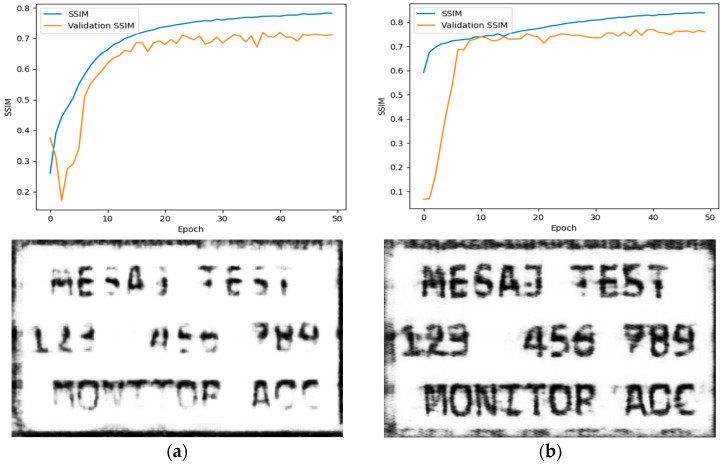
(**a**) Validation SSIM for the classical auto-encoder reached 0.71; the model’s prediction on the test image from Figure 4 is shown below the graph; (**b**) validation SSIM for the auto-encoder with skip connections (U-Net) reached 0.77; the model’s prediction on the test image from Figure 4 is shown below the graph.

Because this paper aims to compare a classical method with a DL method for de-noising TEMPEST video frames, to select the model for further discussion and performance evaluation, 50 epochs of training were employed, and the U-Net architecture was chosen based on the higher validation SSIM. The results can be observed below in Figure 10.

Using 4-fold cross-validation on our dataset, we validated the robustness and generalizability of the auto-encoder. This process also highlighted consistent performance, with minimal fluctuations in accuracy, reinforcing the reliability of the proposed de-noising method.

## 3. Results

The analysis of the proposed methods was initially conducted by calculating the SSIM [18] value both before and after each implemented technique. Furthermore, considering the security perspective associated with TEMPEST imagery, the open-source Tesseract OCR engine [9] was used to assess its accuracy in character recognition from denoised video frames. 

### 3.1. Structural Similarity Index Metric (SSIM)

Many of the evaluation metrics rely on calculating the differences in pixel intensities between a test image and a reference image (e.g., MSE, Mean Absolute Error (MAE), Root Mean Square Error (RMSE)). The SSIM, on the other hand, takes a different approach and is computed based on three fundamental comparison functions that represent key features in an image: luminance comparison Function (5), contrast comparison Function (6), and structure comparison Function (7). The following equations describe these functions:

(5) $$l(x,y)=\frac{2\mu_{x}\mu_{y}+C_{1}}{\mu_{x}^{2}+\mu_{y}^{2}+C_{1}}$$

(6) $$c(x,y)=\frac{2\sigma_{x}\sigma_{y}+C_{2}}{\sigma_{x}^{2}+\sigma_{y}^{2}+C_{2}}$$

(7) $$s\left(x,y\right)=\frac{\sigma_{xy}+C_{3}}{\sigma_{x}\sigma_{y}+C_{3}}$$

where $\mu_{x/y}$ and $\sigma_{x/y}$ are mean intensity and standard deviation of all pixels, respectively, for images x and y, and $C_{1}$, $C_{2}$, and $C_{3}$ are constants meant to ensure stability when the denominator becomes 0. Also, $\sigma_{xy}$ denotes the covariance of the two images. Knowing these, the similarity is computed according to Equation (8):

(8) $$SSIM\left(x,y\right)={\left[l(x,y)\right]}^{\alpha }[{c(x,y)]}^{\beta }{\left[s\left(x,y\right)\right]}^{\gamma }$$

with α > 0, β > 0, γ > 0 denoting the importance of each of the functions. For the present work, α = β = γ = 1, and $C_{3}$ = $\frac{C_{2}}{2}$, which change Equation (9) as follows:

(9) $$SSIM\left(x,y\right)=\frac{\left(2\mu_{x}\mu_{y}+C_{1})({2\sigma }_{xy}+C_{2}\right)}{{(\mu }_{x}^{2}+\mu_{y}^{2}+C_{1})(\sigma_{x}^{2}+\sigma_{y}^{2}+C_{2})}$$

Considering the above theoretical notes, a more efficient approach is to implement the SSIM locally in images and then take the overall mean of the SSIM. Thus, as detailed in [16], an 11 × 11 circular-symmetric Gaussian weighting function was used, and the required metrics calculated locally are described in following equations:

(10) $$\mu_{x}=\sum_{i=1}^{N}w_{i}x_{i}$$

(11) $$\sigma_{x}={\left(\sum_{i=1}^{N}w_{i}{\left(x_{i}-\mu_{x}\right)}^{2}\right)}^{\frac{1}{2}}$$

(12) $$\sigma_{xy}=\sum_{i=1}^{N}w_{i}(x_{i}-\mu_{x})(y_{i}-\mu_{y})$$

where $w_{i}$ is the Gaussian weighting function. The global SSIM value is then computed based on the mean of all the local SSIMs for images X and Y:

(13) $$SIM\left(X,Y\right)=\frac{1}{M}\sum_{j=1}^{M}SSIM(x_{j},y_{j})$$

### 3.2. Performance Evaluation

The similarity between processed noisy video frames and clean ones was studied in the first assessment. To check the algorithms’ robustness, besides testing the image as it was reconstructed from unintended emanations, additional Gaussian noise with increasing sigma values was added. The SSIM value for each circumstance is shown in Table 2.

The application of the proposed methods in the initial test yielded a significant increase in the SSIM from 0.22 to 0.53 and 0.77, respectively. These results offer promising research perspectives both in the direction of classical image processing methods and especially in the direction given by the emergence of DL methods. The tests where extra Gaussian noise is added to the test image demonstrate the robustness of the algorithms, as the SSIM value for the implemented filter remains above 0.4, whereas the auto-encoder model’s performance only starts to decrease after a very high sigma value characterizing the additional noise.

**Figure 11:**
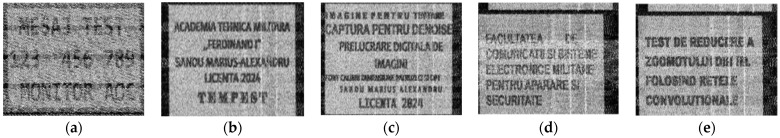
(**a**) Image test #1; (**b**) image test #2; (**c**) image test #3; (**d**) image test #4; (**e**) image test #5.

Considering security concerns, the most important evaluation lies in assessing the amount of private information that could be automatically extracted from the processed video frames using the proposed methods. To better emphasize the obtained results, from the reconstructed images, five of them are presented in Figure 11. The process of reconstruction leads to successfully recognizing approximately 350 characters from different images. As previously mentioned, the OCR engine Tesseract was used, and its predictions’ accuracy was analyzed. Results are shown in Table 3. 

From the OCR results perspective, it becomes evident that while both demonstrate proficiency, the DL method stands out as a superior choice due to the larger number of recovered characters.

Table 4 shows the runtime for the methods discussed and points to the auto-encoder as being the best choice when considering the computational complexity for real-world applications. The adaptive Wiener filter has a bigger de-noising time due to its nature, being based on sliding windows in the spatial domain. It is also well known that applying the filter in the spatial domain to images larger than those used in this paper will increase de-noising time for the AWF. 

## 4. Discussion

This study examines TEMPEST video frames and implements two processing methods for de-noising them in order to increase awareness among users regarding video display unit security breaches and to facilitate video frame reconstruction during the TEMPEST testing of electronic devices. Given the recent advances in artificial intelligence and the necessity for the secure usage of electronic devices in various domains, the proposed methods will significantly enhance defenses against eavesdropping on video display units. Proving the security risks to which an end user might be exposed reinforces the importance of considering countermeasures against electronic equipment eavesdropping. Even if the communication between endpoints is encrypted, processed information could still be compromised by an EM security breach.

Both the adaptive Wiener filter and auto-encoder led to a significant improvement in terms of the average SSIM. Moreover, these methods demonstrated robustness to additional Gaussian noise. Additionally, the study explored recoverable text information from the noisy video frames, which was not feasible with the unprocessed images. The results confirm the anticipated good results of introducing skip connections in the auto-encoder architecture. Training the auto-encoder involved noisy–clean image pairs, but other papers have also studied noise2noise DL models [23]. As for the adaptive filter, a suitable variant has been successfully implemented for the type of data generated by TEMPEST imagery, inspired by the work of J. S. Lim [10], who extensively details the theoretical concept.

It is important to note the similarity between the studied methods. Specifically, the adaptive Wiener filter is a linear approach that modifies the pixels only if local variance is small or applies a skip connection for the pixels where local variance is large. On the other hand, the utilized U-Net architecture represents a non-linear filtering approach of increased complexity, in which the same skip connections pass features before further processing. 

This research work adopts different new approaches regarding the analysis of the unintended electromagnetic emanations generated by electronic devices. Besides the development of the VDU-based process of image reconstruction, another future direction consists of using artificial intelligence in order to colorize the reconstructed frame. Also, a key point regarding our future research work is represented by focusing on the image artifacts that can be observed as horizontal or vertical stripes on the video frames.

Last but not least, Nevertheless, different neural networks could be developed to discover new approaches to the detection and analysis of unintended electromagnetic emissions generated by electronic devices. Applying various neural networks on datasets corresponding to different signals studied in the TEMPEST domain, such as audio, keyboard keystroke signal, USB transfer signal, etc., will reduce the TEMPEST operator’s efforts during the electronic equipment’s testing process.

## Figures and Tables

**Table 1:** Augmentation overview.

Augmentation Applied	Gaussian Blur	Gaussian Noise	Random Contrast	Random Brightness	Histogram Equalization	None
Percentage of Dataset	12.5%	12.5%	12.5%	12.5%	12.5%	37.5%

Random cropping was applied across the entire dataset.

**Table 2:** Evaluation of the proposed methods from the SSIM perspective, with and without extra noise added to intercepted frames.

Test Setup	Avg. SSIM Intercepted Video Frame	Avg. SSIM Adaptive Wiener Filter	Avg. SSIM Auto-Encoder with Skip Connections
No Additional Noise Added	0.22	0.53	0.78
Additional Gaussian Noise Added (σ = 15)	0.14	0.46	0.77
Additional Gaussian Noise Added (σ = 25)	0.07	0.44	0.76
Additional Gaussian Noise Added (σ = 55)	0.01	0.43	0.64

**Table 3:** Performance assessment from an OCR perspective.

Method	OCR Acc. Image Test #1	OCR Acc. Image Test #2	OCR Acc. Image Test #3	OCR Acc. Image Test #4	OCR Acc. Image Test #5	OCR Acc. Total Characters
Adaptive Wiener Filter	50%	64%	58%	41%	59%	54%
Auto-Encoder With Skip Connections	75%	85%	69%	66%	79%	74%

**Table 4:** Computational complexity.

Method	Auto-Encoder with Skip Connections	Adaptive Wiener Filter
De-noising Time	170 ms	4 s

## Data Availability

Data are contained within the article.
